# Supplementary material for: Health Risk Assessment of Heavy Metals in Agricultural Soils Based on Multi-Receptor Modeling Combined with Monte Carlo Simulation
Source: Toxics. 2024 Aug 31;12(9):643. doi: 10.3390/toxics12090643 (PMC11436181; doi:10.3390/toxics12090643)
Supplement: Supplementary file 1 [file toxics-12-00643-s001.zip › toxics-3151741-supplementary.pdf]

Article

Health risk assessment of heavy metals in agricultural soils based on multi-receptor modeling combined with Monte Carlo simulation

Yundong Wu1,#, Yan Xia1,#, Li Mu2,*, Wenjie Liu1,*Qiuying Wang1, Tianyan Su1, Qiu Yang1, Amani Milinga1,

1Center for Eco-Environment Restoration Engineering of Hainan Province, School of Ecology and Environment, Hainan University, Haikou 570228, China. E-mail: liuwj@hainanu.edu.cn; Phone:17733181789;

2Key Laboratory for environmental factors control of Agro-product quality safety (Ministry of Agriculture and Rural Affairs), Tianjin Key Laboratory of Agro-environment and Safe-product, Institute of Agro-environmental Protection, Ministry of Agriculture and Rural Affairs, Tianjin 300191, China

*Corresponding author: Li Mu, Wenjie Liu; Email: muli@caas.cn; liuwj@hainanu.edu.cn;

# These authors contributed equally to this manuscript.

Citation: Wu, Y.; Xia, Y.; Mu, L.; Liu, W.; Wang, Q.; Su, T.; Yang, Q.; Milinga, A.; Zhang, Y.; Huang, Y. Health Risk Assessment of Heavy Metals in Agricultural Soils Based on Multi-Receptor Modeling Combined with Monte Carlo Simulation. *Toxics* **2024**, *12*, x. https://doi.org/10.3390/xxxxx

Academic Editor: Emilio Benfenati

Received: 26 July 2024
Revised: 23 August 2024
Accepted: 27 August 2024
Published: date

Copyright: © 2024 by the authors. Submitted for possible open access publication under the terms and conditions of the Creative Commons Attribution (CC BY) license (https://creativecommons.org/licenses/by/4.0/).

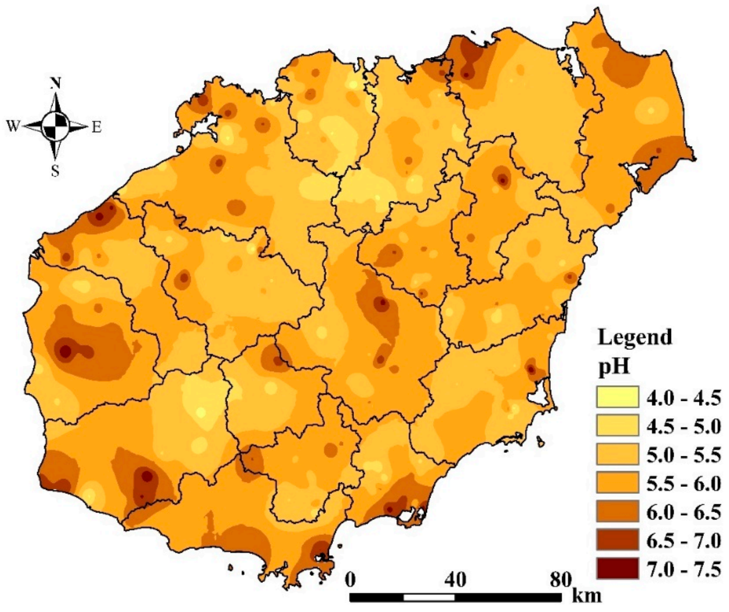

Figure S1. Spatial interpolation of pH on Hainan Island.

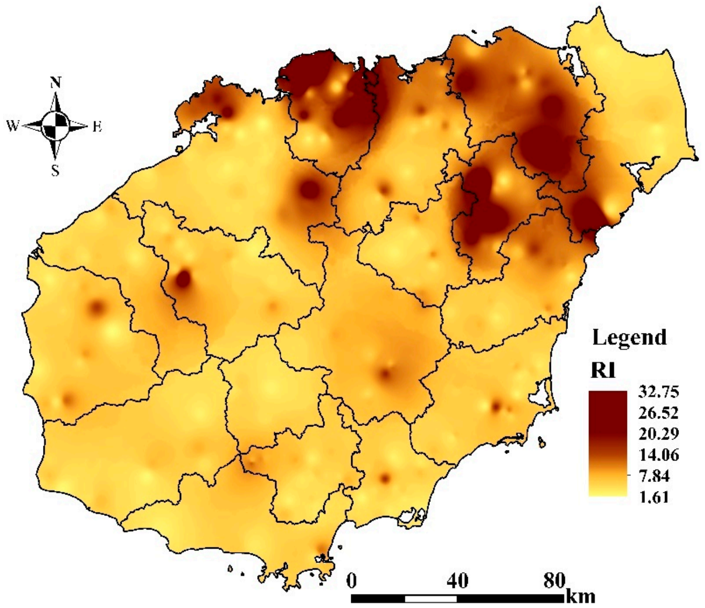

Figure. S2. Spatial interpolation of potential ecological hazard index (RI)

Table S1. Interpretation of total variance of soil PCA.

subassemblies	Initial eigenvalue			Extract the sum of the squares of the loads			Rotational load sum of squares		
	total	Variance %	Cumulative %	total	Variance %	Cumulative %	total	Variance %	Cumulative %
1	2.98	42.65	42.65	2.98	42.66	42.66	2.87	41.02	41.02
2	1.64	23.42	66.08	1.64	23.42	66.08	1.20	17.19	58.21
3	0.85	12.25	78.34	0.86	12.26	78.34	1.04	14.97	73.18
4	0.67	9.60	87.94	0.67	9.61	87.95	1.03	14.76	87.95
5	0.47	6.80	94.75						
6	0.26	3.81	98.57						
7	0.10	1.42	100.00						

Table S2. Statistics of heavy metals in different exposure routes of health risk.

		ADD			HQ		
		Ingest	Dermal	Inlahation	Ingest	Dermal	Inlahation
		Mean	Mean	Mean	Mean	Mean	Mean
Cr	Adults	8.97E-05	1.02E-06	8.47E-07	2.99E-02	3.41E-02	2.96E-02
	Children	8.38E-04	2.34E-06	2.04E-06	2.79E-01	7.82E-02	7.15E-02
Ni	Adults	3.48E-05	3.97E-07	3.29E-07	1.74E-03	7.40E-05	3.65E-03
	Children	3.25E-04	9.10E-07	7.93E-07	1.62E-02	1.68E-04	8.82E-03
Cu	Adults	2.73E-05	3.11E-07	2.58E-07	6.83E-04	2.60E-05	—
	Children	2.55E-04	7.14E-07	6.22E-07	6.37E-03	5.95E-05	—
Zn	Adults	9.67E-05	1.10E-06	9.12E-07	3.22E-04	1.84E-05	—
	Children	9.02E-04	2.53E-06	2.20E-06	3.01E-03	4.21E-05	—
As	Adults	4.62E-06	5.26E-08	4.36E-08	1.54E-02	4.28E-04	1.02E-02
	Children	4.31E-05	1.21E-07	1.05E-07	1.44E-01	9.81E-04	2.45E-02
Pb	Adults	5.04E-05	5.75E-07	4.76E-07	3.60E-02	1.10E-03	—
	Children	4.71E-04	1.32E-06	1.15E-06	3.36E-01	2.52E-03	—
Cd	Adults	2.14E-07	2.44E-09	2.02E-09	2.14E-04	9.76E-05	7.06E-04
	Children	1.99E-06	5.59E-09	4.88E-09	1.99E-03	2.24E-04	1.71E-03

toxics MDPI

Table S3. Individual pollution index (*PI*), Ground Cumulative Index（I_{geo}）and Ecological Risk Index （E_r^i）Pollution Level Classification

Class distribution (%)	Cr	Ni	Cu	Zn	As	Pb	Cd
Individual pollution index (*PI*)							
$PI < 1$	90.56	89.44	92.50	99.17	99.17	98.06	92.22
$1 \leq PI < 2$	9.17	5.56	7.22	0.56	0.83	1.67	6.39
$2 \leq PI < 3$	0.28	5.00	0.28	0.28	0.00	0.28	0.83
$PI \geq 3$	0.00	0.00	0.00	0.00	0.00	0.00	0.56
Geo-accumulation index （I_{geo}）							
$I_{geo} \leq 0$	80.00	80.28	81.67	57.78	96.94	90.28	32.22
$0 < I_{geo} \leq 1$	10.00	6.67	10.00	38.06	2.22	8.89	33.33
$1 < I_{geo} \leq 2$	8.61	3.61	7.78	3.89	0.56	0.83	28.33
$2 < I_{geo} \leq 3$	1.39	8.33	0.56	0.28	0.28	0.00	4.44
$3 < I_{geo} \leq 4$	0.00	1.11	0.00	0.00	0.00	0.00	1.11
$4 < I_{geo} \leq 5$	0.00	0.00	0.00	0.00	0.00	0.00	0.56
$5 < I_{geo}$	0.00	0.00	0.00	0.00	0.00	0.00	0.00
Ecological Risk Index （E_r^i）							
$E_r^i < 40$	100	100	100	100	100	100	100
$40 \leq E_r^i < 80$	0.00	0.00	0.00	0.00	0.00	0.00	0.00
$80 \leq E_r^i < 160$	0.00	0.00	0.00	0.00	0.00	0.00	0.00
$160 \leq E_r^i < 320$	0.00	0.00	0.00	0.00	0.00	0.00	0.00
$E_r^i \geq 320$	0.00	0.00	0.00	0.00	0.00	0.00	0.00

Table S4. Contributions (%) of each factor to soil heavy metals using PMF and APCS/MLR.

HMs	PMF					APCS-MLR					Average			
	F1	F2	F3	F4	R2	F1	F2	F3	F4	R2	F1	F2	F3	F4
Cr	82.70	12.10	2.60	2.70	0.91	74.44	12.77	6.57	6.23	0.84	78.57	12.43	4.58	4.46
Ni	95.00	0.00	1.50	3.50	0.92	79.38	14.25	3.51	2.86	0.90	87.19	7.12	2.51	3.18
Cu	65.00	26.90	2.70	5.40	0.57	72.46	24.49	1.52	1.53	0.75	68.73	25.69	2.11	3.47
Zn	24.80	70.80	0.00	4.30	0.55	31.41	52.79	11.55	4.26	0.72	28.10	61.79	5.77	4.28
As	0.10	0.00	4.30	95.50	0.99	8.13	30.71	14.59	46.57	0.99	4.11	15.35	9.45	71.04
Pb	3.10	78.50	15.20	3.20	0.60	2.67	89.55	6.07	1.70	0.92	2.89	84.03	10.64	2.45
Cd	0.10	11.80	87.30	0.80	0.99	5.32	31.10	61.09	2.48	0.99	2.71	21.45	74.20	1.64
